# Tris(biphenyl-4-yl)arsane

**DOI:** 10.1107/S160053681100211X

**Published:** 2011-01-22

**Authors:** Omar bin Shawkataly, Imthyaz Ahmed Khan, Siti Syaida Sirat, Chin Sing Yeap, Hoong-Kun Fun

**Affiliations:** a Chemical Sciences Programme, School of Distance Education, Universiti Sains Malaysia, 11800 USM, Penang, Malaysia; b X-ray Crystallography Unit, School of Physics, Universiti Sains Malaysia, 11800 USM, Penang, Malaysia

## Abstract

The asymmetric unit of title compound, C_36_H_27_As, contains two crystallographically independent mol­ecules, *A* and *B*, with similar conformations. The two phenyl rings of each biphenyl unit are twisted slightly away from each other with dihedral angles of 6.0 (2), 27.7 (3) and 33.4 (2)° in mol­ecule *A* and 5.7 (3), 27.5 (2) and 33.0 (2)° in mol­ecule *B*. The As-bonded phenyl rings make dihedral angles of 54.9 (2), 76.0 (2) and 88.2 (2),° with each other in mol­ecule *A*, and 60.3 (2), 78.1 (2) and 79.5 (2)° in mol­ecule *B*. In the crystal, the mol­ecules are stacked down the *b* axis. Weak inter­molecular C—H⋯π inter­actions stabilize the crystal structure. The crystal studied was a racemic twin, the refined ratio of twin components being 0.461 (7):0.539 (7).

## Related literature

For structures of related tris­aryl arsane derivatives, see: Cullen *et al.* (1995 ▶); Shawkataly *et al.* (2010*a* ▶,*b* ▶). For the stability of the temperature controller used in the data collection, see: Cosier & Glazer (1986 ▶).
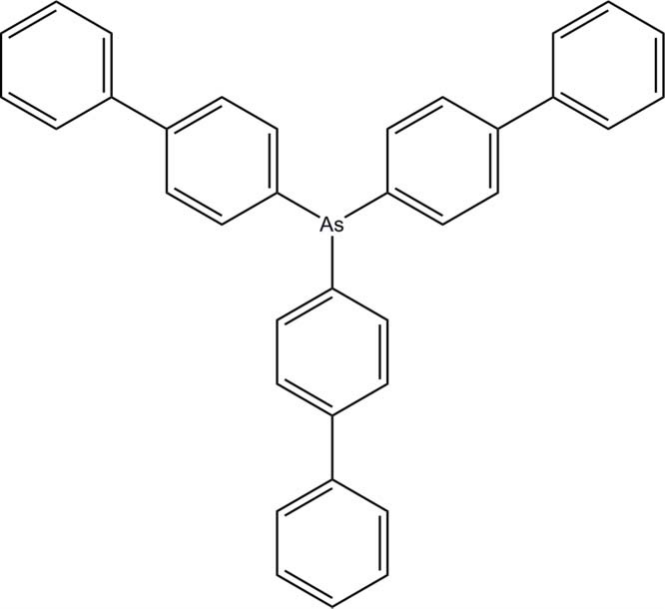


## Experimental

### 

#### Crystal data

C_36_H_27_As
*M* *_r_* = 534.50
Monoclinic, 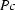

*a* = 17.3718 (19) Å
*b* = 5.9561 (8) Å
*c* = 27.727 (3) Å
β = 114.762 (5)°
*V* = 2605.1 (5) Å^3^
*Z* = 4
Mo *K*α radiation
μ = 1.33 mm^−1^
*T* = 100 K
0.33 × 0.07 × 0.03 mm

#### Data collection

Bruker APEXII DUO CCD area-detector diffractometer
Absorption correction: multi-scan (*SADABS*; Bruker, 2009 ▶) *T*_min_ = 0.671, *T*_max_ = 0.961
23087 measured reflections
11576 independent reflections
8126 reflections with *I* > 2σ(*I*)’
*R*_int_ = 0.057

#### Refinement

*R*[*F* ^2^ > 2σ(*F* ^2^)] = 0.045
*wR*(*F* ^2^) = 0.093
*S* = 1.01
11576 reflections
668 parameters
2 restraints
H-atom parameters constrained
Δρ_max_ = 0.56 e Å^−3^
Δρ_min_ = −0.75 e Å^−3^
Absolute structure: Flack (1983 ▶), 5634 Friedel pairs
Flack parameter: 0.461 (7)

### 

Data collection: *APEX2* (Bruker, 2009 ▶); cell refinement: *SAINT* (Bruker, 2009 ▶); data reduction: *SAINT*; program(s) used to solve structure: *SHELXTL* (Sheldrick, 2008 ▶); program(s) used to refine structure: *SHELXTL*; molecular graphics: *SHELXTL*; software used to prepare material for publication: *SHELXTL* and *PLATON* (Spek, 2009 ▶).

## Supplementary Material

Crystal structure: contains datablocks global, I. DOI: 10.1107/S160053681100211X/ng5092sup1.cif

Structure factors: contains datablocks I. DOI: 10.1107/S160053681100211X/ng5092Isup2.hkl

Additional supplementary materials:  crystallographic information; 3D view; checkCIF report

## Figures and Tables

**Table 1:** Hydrogen-bond geometry (Å, °) *Cg*1, *Cg*2, *Cg*3, *Cg*4, *Cg*5, *Cg*6 and *Cg*7 are the centroids of the C25*B*–C30*B*, C19*B*–C24*B*, C7*B*–C12*B*, C31*A*–C36*A*, C1*A*–C6*A*, C31*B*–C36*B* and C13*B*–C18*B* benzene rings, respectively.

*D*—H⋯*A*	*D*—H	H⋯*A*	*D*⋯*A*	*D*—H⋯*A*
C4*B*—H4*BA*⋯*Cg*1^i^	0.93	2.77	3.583 (6)	147
C9*A*—H9*A*⋯*Cg*2^ii^	0.93	2.99	3.872 (6)	160
C11*A*—H11*A*⋯*Cg*3^iii^	0.93	2.88	3.723 (7)	153
C13*B*—H13*B*⋯*Cg*4	0.93	2.79	3.596 (5)	147
C14*A*—H14*A*⋯*Cg*5^i^	0.93	2.93	3.834 (5)	172
C24*A*—H24*A*⋯*Cg*6^iv^	0.93	2.70	3.582 (6)	160
C28*B*—H28*B*⋯*Cg*7^v^	0.93	2.56	3.313 (6)	139
C36*A*—H36*A*⋯*Cg*3^vi^	0.93	2.96	3.651 (6)	133

Symmetry codes: (i) 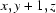; (ii) 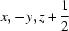; (iii) 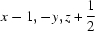; (iv) 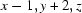; (v) 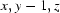; (vi) 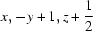.

**Fig. 1.**
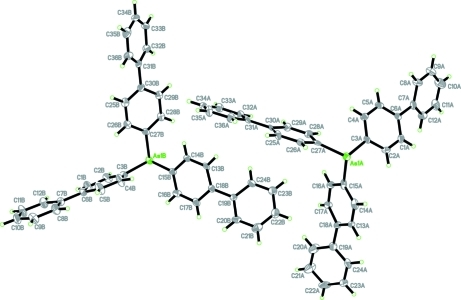
The molecular structure of the title compound with 50% probability ellipsoids for non-H atoms.

**Fig. 2.**
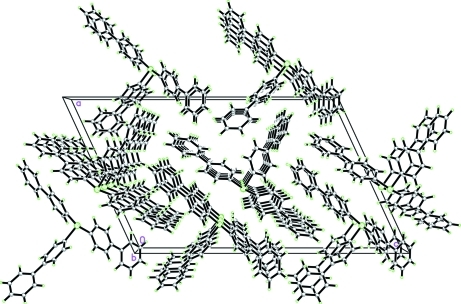
The crystal packing of the title compound, viewed down the b axis, showing the molecules stacked down the b axis.

## supplementary crystallographic information

### Comment

Trisaryl arsanes have been used in the synthesis of Osmium and Ruthenium cluster derivatives (Cullen *et al.*, 1995; Shawkataly *et al.*, 2010*a*, *b*). In continuation of our work on trisaryl arsanes, we report the synthesis and structure of title compound.

The asymmetric unit consists of two crystallographically independent molecules of trisaryl arsane, *A* and *B*, with similar conformation (Fig. 1). Both phenyl rings of biphenyl are slightly twisted from each other with the dihedral angles of 33.4 (2), 27.7 (3) and 6.0 (2)° in molecule *A* whereas these angles are 5.7 (3), 27.5 (2) and 33.0 (2)° in molecule *B*. The arsane-substituted phenyl rings make dihedral angles of 88.2 (2), 54.9 (2) and 76.0 (2)° with each other in molecule *A* whereas 79.5 (2), 78.1 (2) and 60.3 (2)° in molecule *B*. In the crystal packing, the molecules are stacked down the *b* axis (Fig 2). Weak intermolecular C—H···π interactions stabilize the crystal structure (Table 1).

### Experimental

All manipulations were performed under a dry oxygen-free nitrogen atmosphere using standard Schlenk techniques. All solvents were dried over sodium and distilled from sodium benzophenone ketyl under dry oxygen free nitrogen. Tris(1-biphenyl)arsane was prepared from arsenic trichloride and biphenyl magnesiumbromide in tetrahydrofuran. Crystals suitable for X-ray diffraction were grown by slow solvent / solvent diffusion of CH_3_OH into CHCl_3_.

### Refinement

All hydrogen atoms were positioned geometrically and refined using a riding model with C—H = 0.93 Å and *U*_iso_(H) = 1.2 *U*_eq_(C). The crystal studied is a racemic twin with the refined ratio of twin components being 0.461 (7):0.539 (7). Fourteen outliers reflections (-2 4 -6, -4 4-3, 1 0 0, 6 4 -9, -6 4 10, 9 0 -18, 2 4 6, -3 4 -4, -9 0 18, 4 4 3, 3 4 4, 10 0 -14, 4 3 -15 and -1 4 -7) were omitted from the final refinement.

### Crystal data

**Table:**

C_36_H_27_As	*F*(000) = 1104
*M**_r_* = 534.50	*D*_x_ = 1.363 Mg m^−^^3^
Monoclinic, *P**c*	Mo *K*α radiation, λ = 0.71073 Å
Hall symbol: P -2yc	Cell parameters from 4340 reflections
*a* = 17.3718 (19) Å	θ = 2.4–26.2°
*b* = 5.9561 (8) Å	µ = 1.33 mm^−^^1^
*c* = 27.727 (3) Å	*T* = 100 K
β = 114.762 (5)°	Needle, colourless
*V* = 2605.1 (5) Å^3^	0.33 × 0.07 × 0.03 mm
*Z* = 4	

### Data collection

**Table:**

Bruker APEXII DUO CCD area-detector diffractometer	11576 independent reflections
Radiation source: fine-focus sealed tube	8126 reflections with *I* > 2σ(*I*)'
graphite	*R*_int_ = 0.057
φ and ω scans	θ_max_ = 27.5°, θ_min_ = 1.6°
Absorption correction: multi-scan (*SADABS*; Bruker, 2009)	*h* = −22→22
*T*_min_ = 0.671, *T*_max_ = 0.961	*k* = −7→7
23087 measured reflections	*l* = −35→36

### Refinement

**Table:**

Refinement on *F*^2^	Secondary atom site location: difference Fourier map
Least-squares matrix: full	Hydrogen site location: inferred from neighbouring sites
*R*[*F*^2^ > 2σ(*F*^2^)] = 0.045	H-atom parameters constrained
*wR*(*F*^2^) = 0.093	*w* = 1/[σ^2^(*F*_o_^2^) + (0.0318*P*)^2^] where *P* = (*F*_o_^2^ + 2*F*_c_^2^)/3
*S* = 1.01	(Δ/σ)_max_ < 0.001
11576 reflections	Δρ_max_ = 0.56 e Å^−^^3^
668 parameters	Δρ_min_ = −0.75 e Å^−^^3^
2 restraints	Absolute structure: Flack (1983), 5634 Friedel pairs
Primary atom site location: structure-invariant direct methods	Flack parameter: 0.461 (7)

### Special details

**Table:**

Experimental. The crystal was placed in the cold stream of an Oxford Cryosystems Cobra open-flow nitrogen cryostat (Cosier & Glazer, 1986) operating at 100.0 (1) K.
Geometry. All esds (except the esd in the dihedral angle between two l.s. planes) are estimated using the full covariance matrix. The cell esds are taken into account individually in the estimation of esds in distances, angles and torsion angles; correlations between esds in cell parameters are only used when they are defined by crystal symmetry. An approximate (isotropic) treatment of cell esds is used for estimating esds involving l.s. planes.
Refinement. Refinement of F^2^ against ALL reflections. The weighted R-factor wR and goodness of fit S are based on F^2^, conventional R-factors R are based on F, with F set to zero for negative F^2^. The threshold expression of F^2^ > 2sigma(F^2^) is used only for calculating R-factors(gt) etc. and is not relevant to the choice of reflections for refinement. R-factors based on F^2^ are statistically about twice as large as those based on F, and R- factors based on ALL data will be even larger.

### Fractional atomic coordinates and isotropic or equivalent isotropic displacement parameters (Å^2^)

**Table:**

	*x*	*y*	*z*	*U*_iso_*/*U*_eq_	
As1A	0.194411 (19)	0.60795 (9)	0.860597 (14)	0.01986 (13)	
C1A	0.0224 (3)	0.2913 (8)	0.90946 (16)	0.0243 (10)	
H1A	−0.0305	0.3282	0.9081	0.029*	
C2A	0.0615 (3)	0.4369 (8)	0.88744 (16)	0.0239 (11)	
H2A	0.0345	0.5701	0.8719	0.029*	
C3A	0.1404 (3)	0.3872 (9)	0.88811 (19)	0.0193 (13)	
C4A	0.1800 (3)	0.1885 (8)	0.91370 (15)	0.0215 (10)	
H4A	0.2335	0.1527	0.9159	0.026*	
C5A	0.1410 (3)	0.0459 (7)	0.93548 (15)	0.0212 (10)	
H5A	0.1687	−0.0850	0.9520	0.025*	
C6A	0.0607 (3)	0.0928 (8)	0.93336 (15)	0.0176 (9)	
C7A	0.0188 (3)	−0.0670 (8)	0.95564 (16)	0.0237 (10)	
C8A	0.0649 (3)	−0.2009 (8)	0.99967 (16)	0.0273 (10)	
H8A	0.1236	−0.1876	1.0158	0.033*	
C9A	0.0260 (3)	−0.3526 (8)	1.02006 (18)	0.0346 (12)	
H9A	0.0585	−0.4393	1.0495	0.042*	
C10A	−0.0613 (3)	−0.3752 (9)	0.99666 (19)	0.0397 (13)	
H10A	−0.0882	−0.4741	1.0106	0.048*	
C11A	−0.1079 (4)	−0.2480 (10)	0.9522 (2)	0.0341 (14)	
H11A	−0.1664	−0.2653	0.9357	0.041*	
C12A	−0.0698 (3)	−0.0975 (9)	0.9320 (2)	0.0288 (13)	
H12A	−0.1028	−0.0137	0.9022	0.035*	
C13A	−0.0044 (3)	0.9509 (8)	0.73934 (16)	0.0196 (10)	
H13A	−0.0294	1.0908	0.7370	0.024*	
C14A	0.0582 (2)	0.8804 (8)	0.78741 (15)	0.0231 (9)	
H14A	0.0737	0.9741	0.8168	0.028*	
C15A	0.0985 (3)	0.6743 (8)	0.79307 (18)	0.0194 (11)	
C16A	0.0697 (3)	0.5329 (8)	0.74858 (16)	0.0224 (10)	
H16A	0.0938	0.3914	0.7512	0.027*	
C17A	0.0061 (3)	0.6006 (8)	0.70083 (16)	0.0225 (9)	
H17A	−0.0130	0.5015	0.6723	0.027*	
C18A	−0.0302 (3)	0.8138 (8)	0.69438 (16)	0.0200 (9)	
C19A	−0.0926 (2)	0.8984 (8)	0.64179 (15)	0.0220 (9)	
C20A	−0.0917 (3)	0.8198 (10)	0.59537 (18)	0.0487 (16)	
H20A	−0.0529	0.7098	0.5966	0.058*	
C21A	−0.1487 (4)	0.9040 (11)	0.54635 (19)	0.063 (2)	
H21A	−0.1472	0.8511	0.5152	0.075*	
C22A	−0.2074 (3)	1.0657 (10)	0.54385 (18)	0.0485 (16)	
H22A	−0.2457	1.1207	0.5112	0.058*	
C23A	−0.2088 (3)	1.1427 (8)	0.58916 (17)	0.0283 (11)	
H23A	−0.2485	1.2503	0.5877	0.034*	
C24A	−0.1514 (3)	1.0629 (8)	0.63790 (17)	0.0275 (11)	
H24A	−0.1523	1.1210	0.6688	0.033*	
C25A	0.3887 (3)	0.4234 (8)	0.81806 (15)	0.0216 (10)	
H25A	0.4378	0.4963	0.8208	0.026*	
C26A	0.3378 (3)	0.5241 (8)	0.83881 (15)	0.0216 (10)	
H26A	0.3535	0.6633	0.8552	0.026*	
C27A	0.2637 (3)	0.4242 (8)	0.83604 (16)	0.0189 (11)	
C28A	0.2452 (3)	0.2124 (8)	0.81276 (16)	0.0212 (10)	
H28A	0.1971	0.1380	0.8111	0.025*	
C29A	0.2958 (3)	0.1095 (8)	0.79215 (17)	0.0216 (10)	
H29A	0.2815	−0.0329	0.7773	0.026*	
C30A	0.3684 (3)	0.2149 (8)	0.79310 (15)	0.0183 (9)	
C31A	0.4232 (2)	0.1064 (8)	0.77069 (14)	0.0181 (9)	
C32A	0.3995 (3)	−0.0934 (8)	0.74170 (16)	0.0230 (10)	
H32A	0.3476	−0.1581	0.7358	0.028*	
C33A	0.4514 (3)	−0.1994 (9)	0.72121 (18)	0.0281 (12)	
H33A	0.4349	−0.3348	0.7031	0.034*	
C34A	0.5278 (3)	−0.1000 (8)	0.72826 (15)	0.0245 (10)	
H34A	0.5625	−0.1673	0.7144	0.029*	
C35A	0.5515 (3)	0.0987 (8)	0.75584 (16)	0.0279 (10)	
H35A	0.6023	0.1661	0.7603	0.033*	
C36A	0.5009 (3)	0.2000 (8)	0.77705 (16)	0.0249 (10)	
H36A	0.5189	0.3332	0.7960	0.030*	
As1B	0.42591 (2)	−0.11085 (9)	0.505294 (14)	0.02100 (13)	
C1B	0.5352 (3)	0.1100 (9)	0.40554 (17)	0.0323 (11)	
H1BA	0.5353	0.0548	0.3742	0.039*	
C2B	0.4891 (3)	−0.0029 (8)	0.42850 (16)	0.0280 (11)	
H2BA	0.4597	−0.1328	0.4127	0.034*	
C3B	0.4866 (3)	0.0761 (9)	0.4747 (2)	0.0210 (12)	
C4B	0.5299 (3)	0.2709 (8)	0.49691 (18)	0.0355 (13)	
H4BA	0.5277	0.3291	0.5274	0.043*	
C6B	0.5810 (3)	0.3018 (8)	0.42787 (17)	0.0195 (10)	
C7B	0.6335 (3)	0.4195 (8)	0.40412 (15)	0.0202 (9)	
C8B	0.6727 (3)	0.6205 (9)	0.4241 (2)	0.0333 (15)	
H8BA	0.6664	0.6863	0.4526	0.040*	
C9B	0.7224 (4)	0.7278 (11)	0.4019 (2)	0.0391 (15)	
H9BA	0.7491	0.8629	0.4161	0.047*	
C10B	0.7315 (3)	0.6348 (10)	0.35979 (18)	0.0393 (13)	
H10B	0.7643	0.7062	0.3452	0.047*	
C11B	0.6919 (3)	0.4344 (9)	0.33885 (18)	0.0390 (13)	
H11B	0.6979	0.3704	0.3100	0.047*	
C12B	0.6430 (3)	0.3281 (9)	0.36081 (17)	0.0336 (12)	
H12B	0.6162	0.1934	0.3463	0.040*	
C13B	0.3642 (2)	0.2341 (7)	0.61791 (15)	0.0207 (10)	
H13B	0.3783	0.2222	0.6541	0.025*	
C14B	0.4049 (2)	0.0969 (8)	0.59546 (14)	0.0205 (9)	
H14B	0.4450	−0.0065	0.6165	0.025*	
C15B	0.3863 (3)	0.1127 (8)	0.54223 (17)	0.0201 (11)	
C16B	0.3278 (3)	0.2738 (8)	0.51198 (16)	0.0238 (10)	
H16B	0.3165	0.2906	0.4763	0.029*	
C17B	0.2864 (3)	0.4092 (8)	0.53410 (16)	0.0238 (10)	
H17B	0.2472	0.5145	0.5130	0.029*	
C18B	0.3027 (3)	0.3896 (8)	0.58774 (17)	0.0187 (10)	
C19B	0.2561 (3)	0.5226 (8)	0.61262 (16)	0.0191 (10)	
C20B	0.2213 (3)	0.7349 (8)	0.59293 (17)	0.0265 (11)	
H20B	0.2284	0.7958	0.5642	0.032*	
C21B	0.1770 (3)	0.8532 (8)	0.61548 (18)	0.0315 (11)	
H21B	0.1543	0.9929	0.6020	0.038*	
C22B	0.1660 (3)	0.7640 (9)	0.6586 (2)	0.0331 (13)	
H22B	0.1355	0.8438	0.6736	0.040*	
C23B	0.2001 (3)	0.5589 (8)	0.67895 (18)	0.0292 (11)	
H23B	0.1936	0.5005	0.7081	0.035*	
C24B	0.2442 (3)	0.4392 (7)	0.65571 (16)	0.0219 (10)	
H24B	0.2664	0.2993	0.6694	0.026*	
C25B	0.6718 (3)	−0.3161 (8)	0.61583 (16)	0.0244 (10)	
H25B	0.7279	−0.2926	0.6215	0.029*	
C26B	0.6075 (3)	−0.2017 (7)	0.57495 (16)	0.0219 (9)	
H26B	0.6207	−0.1031	0.5535	0.026*	
C27B	0.5232 (3)	−0.2357 (8)	0.56627 (18)	0.0201 (11)	
C28B	0.5052 (2)	−0.3878 (7)	0.59828 (14)	0.0194 (9)	
H28B	0.4492	−0.4162	0.5920	0.023*	
C29B	0.5700 (3)	−0.4972 (8)	0.63944 (16)	0.0208 (10)	
H29B	0.5569	−0.5937	0.6613	0.025*	
C30B	0.6540 (3)	−0.4650 (8)	0.64851 (18)	0.0215 (11)	
C31B	0.7221 (3)	−0.5891 (8)	0.69188 (15)	0.0209 (9)	
C32B	0.7091 (3)	−0.8021 (8)	0.70683 (16)	0.0267 (10)	
H32B	0.6574	−0.8733	0.6881	0.032*	
C33B	0.7726 (3)	−0.9123 (9)	0.74970 (17)	0.0325 (12)	
H33B	0.7627	−1.0553	0.7593	0.039*	
C34B	0.8492 (3)	−0.8102 (10)	0.77748 (17)	0.0338 (12)	
H34B	0.8912	−0.8830	0.8061	0.041*	
C35B	0.8638 (3)	−0.5993 (10)	0.76277 (17)	0.0348 (12)	
H35B	0.9159	−0.5299	0.7812	0.042*	
C36B	0.8010 (3)	−0.4914 (9)	0.72080 (17)	0.0272 (11)	
H36B	0.8117	−0.3486	0.7114	0.033*	
C56B	0.5767 (3)	0.3802 (9)	0.47409 (19)	0.0343 (12)	
H56A	0.6061	0.5099	0.4901	0.041*	

### Atomic displacement parameters (Å^2^)

**Table:**

	*U*^11^	*U*^22^	*U*^33^	*U*^12^	*U*^13^	*U*^23^
As1A	0.0202 (3)	0.0222 (3)	0.0152 (3)	−0.0001 (3)	0.0055 (2)	−0.0024 (3)
C1A	0.022 (2)	0.031 (3)	0.021 (2)	0.006 (2)	0.010 (2)	0.003 (2)
C2A	0.022 (2)	0.027 (3)	0.021 (2)	0.008 (2)	0.0072 (18)	0.0036 (19)
C3A	0.021 (3)	0.026 (3)	0.010 (2)	−0.003 (2)	0.006 (2)	−0.005 (2)
C4A	0.018 (2)	0.026 (3)	0.018 (2)	0.0065 (19)	0.0052 (18)	0.0014 (19)
C5A	0.027 (2)	0.020 (3)	0.012 (2)	0.0010 (19)	0.0043 (18)	−0.0027 (17)
C6A	0.020 (2)	0.020 (3)	0.010 (2)	−0.001 (2)	0.0040 (17)	−0.0006 (19)
C7A	0.025 (2)	0.025 (3)	0.022 (2)	−0.002 (2)	0.0112 (19)	−0.002 (2)
C8A	0.027 (2)	0.028 (3)	0.020 (2)	−0.001 (2)	0.0039 (19)	0.002 (2)
C9A	0.038 (3)	0.035 (3)	0.028 (2)	0.000 (2)	0.011 (2)	0.009 (2)
C10A	0.043 (3)	0.040 (3)	0.038 (3)	−0.006 (3)	0.018 (2)	0.008 (3)
C11A	0.029 (3)	0.042 (4)	0.034 (3)	−0.012 (3)	0.016 (2)	0.001 (3)
C12A	0.019 (2)	0.042 (4)	0.021 (2)	0.001 (3)	0.004 (2)	0.003 (2)
C13A	0.024 (2)	0.018 (3)	0.020 (2)	0.004 (2)	0.012 (2)	0.003 (2)
C14A	0.026 (2)	0.025 (3)	0.021 (2)	−0.008 (2)	0.0121 (18)	−0.009 (2)
C15A	0.021 (2)	0.020 (3)	0.022 (2)	−0.004 (2)	0.014 (2)	−0.003 (2)
C16A	0.024 (2)	0.021 (2)	0.022 (2)	0.0019 (19)	0.0093 (19)	0.0019 (19)
C17A	0.030 (2)	0.020 (2)	0.018 (2)	0.000 (2)	0.0109 (18)	−0.006 (2)
C18A	0.019 (2)	0.019 (2)	0.021 (2)	−0.0030 (18)	0.0086 (19)	−0.0041 (19)
C19A	0.021 (2)	0.025 (2)	0.019 (2)	0.003 (2)	0.0061 (17)	0.001 (2)
C20A	0.065 (4)	0.054 (4)	0.021 (2)	0.035 (3)	0.012 (2)	−0.002 (2)
C21A	0.083 (4)	0.076 (5)	0.019 (3)	0.048 (4)	0.011 (3)	−0.001 (3)
C22A	0.055 (3)	0.058 (4)	0.017 (2)	0.023 (3)	0.000 (2)	0.004 (3)
C23A	0.025 (2)	0.033 (3)	0.026 (2)	0.008 (2)	0.010 (2)	0.005 (2)
C24A	0.030 (3)	0.033 (3)	0.021 (2)	−0.001 (2)	0.011 (2)	0.000 (2)
C25A	0.020 (2)	0.030 (3)	0.016 (2)	−0.001 (2)	0.0076 (17)	0.0003 (19)
C26A	0.022 (2)	0.022 (3)	0.015 (2)	−0.0044 (19)	0.0025 (18)	−0.0039 (19)
C27A	0.022 (3)	0.021 (3)	0.008 (2)	−0.001 (2)	0.0004 (19)	0.0051 (19)
C28A	0.020 (2)	0.023 (3)	0.020 (2)	−0.005 (2)	0.0087 (19)	−0.003 (2)
C29A	0.024 (2)	0.020 (2)	0.024 (3)	0.002 (2)	0.013 (2)	0.001 (2)
C30A	0.021 (2)	0.024 (3)	0.0082 (19)	0.006 (2)	0.0052 (17)	0.0048 (19)
C31A	0.021 (2)	0.021 (2)	0.0103 (18)	0.000 (2)	0.0048 (16)	0.0037 (19)
C32A	0.028 (2)	0.020 (3)	0.023 (2)	0.000 (2)	0.013 (2)	0.001 (2)
C33A	0.033 (3)	0.031 (3)	0.019 (2)	0.000 (2)	0.009 (2)	−0.006 (2)
C34A	0.025 (2)	0.035 (3)	0.016 (2)	0.007 (2)	0.0110 (18)	0.004 (2)
C35A	0.025 (2)	0.041 (3)	0.023 (2)	−0.005 (2)	0.0146 (19)	−0.004 (2)
C36A	0.024 (2)	0.032 (3)	0.021 (2)	0.000 (2)	0.0116 (19)	−0.004 (2)
As1B	0.0238 (3)	0.0237 (3)	0.0139 (2)	−0.0034 (3)	0.0064 (2)	−0.0017 (3)
C1B	0.048 (3)	0.034 (3)	0.021 (2)	−0.014 (3)	0.020 (2)	−0.009 (2)
C2B	0.036 (3)	0.028 (3)	0.018 (2)	−0.011 (2)	0.009 (2)	−0.010 (2)
C3B	0.024 (3)	0.021 (3)	0.016 (3)	0.004 (2)	0.006 (2)	0.003 (2)
C4B	0.069 (4)	0.026 (3)	0.023 (2)	−0.016 (3)	0.031 (3)	−0.015 (2)
C6B	0.024 (2)	0.023 (3)	0.013 (2)	0.001 (2)	0.0090 (19)	0.001 (2)
C7B	0.021 (2)	0.024 (3)	0.014 (2)	0.001 (2)	0.0065 (17)	0.0011 (19)
C8B	0.044 (4)	0.034 (4)	0.028 (3)	−0.008 (3)	0.021 (3)	−0.005 (3)
C9B	0.041 (3)	0.044 (4)	0.032 (3)	−0.005 (3)	0.015 (3)	0.007 (3)
C10B	0.033 (3)	0.056 (4)	0.032 (3)	0.003 (3)	0.016 (2)	0.014 (3)
C11B	0.048 (3)	0.052 (4)	0.024 (2)	−0.005 (3)	0.022 (2)	−0.001 (2)
C12B	0.041 (3)	0.039 (3)	0.027 (2)	−0.003 (3)	0.020 (2)	−0.004 (2)
C13B	0.026 (2)	0.025 (3)	0.0091 (19)	0.002 (2)	0.0049 (17)	0.0058 (18)
C14B	0.021 (2)	0.026 (2)	0.0121 (18)	0.002 (2)	0.0037 (16)	0.0028 (19)
C15B	0.017 (2)	0.026 (3)	0.014 (2)	−0.005 (2)	0.0028 (19)	−0.002 (2)
C16B	0.027 (2)	0.027 (3)	0.014 (2)	−0.006 (2)	0.0055 (19)	−0.002 (2)
C17B	0.023 (2)	0.026 (3)	0.018 (2)	−0.002 (2)	0.0034 (18)	0.005 (2)
C18B	0.015 (2)	0.020 (2)	0.020 (2)	−0.003 (2)	0.0070 (18)	−0.001 (2)
C19B	0.016 (2)	0.022 (3)	0.018 (2)	−0.0008 (19)	0.0058 (18)	−0.0010 (19)
C20B	0.031 (3)	0.022 (3)	0.025 (2)	−0.001 (2)	0.010 (2)	0.003 (2)
C21B	0.033 (3)	0.025 (3)	0.033 (3)	0.005 (2)	0.012 (2)	−0.002 (2)
C22B	0.033 (3)	0.042 (4)	0.029 (3)	0.003 (3)	0.018 (2)	−0.001 (3)
C23B	0.028 (2)	0.035 (3)	0.027 (2)	−0.001 (2)	0.014 (2)	0.000 (2)
C24B	0.024 (2)	0.021 (3)	0.022 (2)	0.0000 (19)	0.0108 (19)	0.0031 (19)
C25B	0.023 (2)	0.024 (3)	0.026 (2)	−0.002 (2)	0.0107 (19)	−0.004 (2)
C26B	0.027 (2)	0.020 (2)	0.020 (2)	−0.004 (2)	0.0112 (19)	−0.0031 (19)
C27B	0.025 (2)	0.020 (3)	0.016 (2)	0.000 (2)	0.009 (2)	−0.002 (2)
C28B	0.022 (2)	0.017 (2)	0.0171 (19)	−0.006 (2)	0.0051 (16)	−0.0068 (19)
C29B	0.027 (2)	0.017 (2)	0.022 (2)	−0.0008 (19)	0.014 (2)	−0.0042 (19)
C30B	0.022 (2)	0.019 (3)	0.021 (2)	0.000 (2)	0.006 (2)	−0.002 (2)
C31B	0.022 (2)	0.026 (3)	0.015 (2)	0.005 (2)	0.0089 (18)	−0.004 (2)
C32B	0.030 (2)	0.030 (3)	0.024 (2)	0.002 (2)	0.015 (2)	0.000 (2)
C33B	0.043 (3)	0.036 (3)	0.028 (2)	0.016 (3)	0.023 (2)	0.010 (2)
C34B	0.032 (3)	0.052 (4)	0.020 (2)	0.019 (3)	0.014 (2)	0.007 (2)
C35B	0.027 (3)	0.051 (4)	0.028 (2)	0.000 (3)	0.013 (2)	−0.014 (3)
C36B	0.027 (3)	0.026 (3)	0.025 (2)	0.004 (2)	0.008 (2)	−0.005 (2)
C56B	0.053 (3)	0.024 (3)	0.037 (3)	−0.018 (3)	0.030 (3)	−0.010 (2)

### Geometric parameters (Å, °)

**Table:**

As1A—C3A	1.947 (5)	As1B—C3B	1.954 (5)
As1A—C27A	1.949 (5)	As1B—C27B	1.968 (5)
As1A—C15A	1.955 (5)	As1B—C15B	1.971 (5)
C1A—C6A	1.382 (6)	C1B—C6B	1.381 (6)
C1A—C2A	1.391 (6)	C1B—C2B	1.387 (6)
C1A—H1A	0.9300	C1B—H1BA	0.9300
C2A—C3A	1.394 (6)	C2B—C3B	1.383 (6)
C2A—H2A	0.9300	C2B—H2BA	0.9300
C3A—C4A	1.403 (6)	C3B—C4B	1.380 (7)
C4A—C5A	1.373 (6)	C4B—C56B	1.383 (6)
C4A—H4A	0.9300	C4B—H4BA	0.9300
C5A—C6A	1.400 (6)	C6B—C56B	1.395 (6)
C5A—H5A	0.9300	C6B—C7B	1.503 (6)
C6A—C7A	1.482 (6)	C7B—C8B	1.374 (7)
C7A—C8A	1.396 (6)	C7B—C12B	1.390 (6)
C7A—C12A	1.409 (6)	C8B—C9B	1.405 (7)
C8A—C9A	1.383 (6)	C8B—H8BA	0.9300
C8A—H8A	0.9300	C9B—C10B	1.360 (7)
C9A—C10A	1.384 (6)	C9B—H9BA	0.9300
C9A—H9A	0.9300	C10B—C11B	1.379 (7)
C10A—C11A	1.383 (7)	C10B—H10B	0.9300
C10A—H10A	0.9300	C11B—C12B	1.387 (6)
C11A—C12A	1.365 (7)	C11B—H11B	0.9300
C11A—H11A	0.9300	C12B—H12B	0.9300
C12A—H12A	0.9300	C13B—C14B	1.387 (5)
C13A—C14A	1.386 (6)	C13B—C18B	1.396 (6)
C13A—C18A	1.398 (6)	C13B—H13B	0.9300
C13A—H13A	0.9300	C14B—C15B	1.376 (6)
C14A—C15A	1.390 (6)	C14B—H14B	0.9300
C14A—H14A	0.9300	C15B—C16B	1.393 (6)
C15A—C16A	1.401 (6)	C16B—C17B	1.384 (6)
C16A—C17A	1.383 (5)	C16B—H16B	0.9300
C16A—H16A	0.9300	C17B—C18B	1.397 (5)
C17A—C18A	1.395 (6)	C17B—H17B	0.9300
C17A—H17A	0.9300	C18B—C19B	1.493 (6)
C18A—C19A	1.494 (5)	C19B—C24B	1.387 (5)
C19A—C20A	1.376 (6)	C19B—C20B	1.409 (6)
C19A—C24A	1.387 (6)	C20B—C21B	1.372 (6)
C20A—C21A	1.397 (7)	C20B—H20B	0.9300
C20A—H20A	0.9300	C21B—C22B	1.391 (7)
C21A—C22A	1.384 (7)	C21B—H21B	0.9300
C21A—H21A	0.9300	C22B—C23B	1.372 (7)
C22A—C23A	1.347 (6)	C22B—H22B	0.9300
C22A—H22A	0.9300	C23B—C24B	1.387 (6)
C23A—C24A	1.385 (6)	C23B—H23B	0.9300
C23A—H23A	0.9300	C24B—H24B	0.9300
C24A—H24A	0.9300	C25B—C26B	1.391 (6)
C25A—C26A	1.378 (6)	C25B—C30B	1.392 (6)
C25A—C30A	1.393 (6)	C25B—H25B	0.9300
C25A—H25A	0.9300	C26B—C27B	1.397 (6)
C26A—C27A	1.391 (6)	C26B—H26B	0.9300
C26A—H26A	0.9300	C27B—C28B	1.392 (6)
C27A—C28A	1.392 (6)	C28B—C29B	1.384 (6)
C28A—C29A	1.378 (6)	C28B—H28B	0.9300
C28A—H28A	0.9300	C29B—C30B	1.387 (6)
C29A—C30A	1.399 (6)	C29B—H29B	0.9300
C29A—H29A	0.9300	C30B—C31B	1.483 (6)
C30A—C31A	1.484 (6)	C31B—C32B	1.382 (6)
C31A—C32A	1.398 (6)	C31B—C36B	1.394 (6)
C31A—C36A	1.402 (6)	C32B—C33B	1.401 (6)
C32A—C33A	1.401 (6)	C32B—H32B	0.9300
C32A—H32A	0.9300	C33B—C34B	1.370 (7)
C33A—C34A	1.390 (6)	C33B—H33B	0.9300
C33A—H33A	0.9300	C34B—C35B	1.376 (7)
C34A—C35A	1.375 (6)	C34B—H34B	0.9300
C34A—H34A	0.9300	C35B—C36B	1.376 (6)
C35A—C36A	1.383 (6)	C35B—H35B	0.9300
C35A—H35A	0.9300	C36B—H36B	0.9300
C36A—H36A	0.9300	C56B—H56A	0.9300
			
C3A—As1A—C27A	103.1 (2)	C3B—As1B—C27B	99.4 (2)
C3A—As1A—C15A	98.9 (2)	C3B—As1B—C15B	102.1 (2)
C27A—As1A—C15A	100.48 (18)	C27B—As1B—C15B	99.78 (19)
C6A—C1A—C2A	121.2 (4)	C6B—C1B—C2B	122.0 (4)
C6A—C1A—H1A	119.4	C6B—C1B—H1BA	119.0
C2A—C1A—H1A	119.4	C2B—C1B—H1BA	119.0
C1A—C2A—C3A	121.3 (4)	C3B—C2B—C1B	120.6 (5)
C1A—C2A—H2A	119.3	C3B—C2B—H2BA	119.7
C3A—C2A—H2A	119.3	C1B—C2B—H2BA	119.7
C2A—C3A—C4A	117.2 (5)	C4B—C3B—C2B	118.4 (5)
C2A—C3A—As1A	118.9 (4)	C4B—C3B—As1B	125.3 (4)
C4A—C3A—As1A	123.6 (4)	C2B—C3B—As1B	116.1 (4)
C5A—C4A—C3A	121.1 (4)	C3B—C4B—C56B	120.4 (4)
C5A—C4A—H4A	119.5	C3B—C4B—H4BA	119.8
C3A—C4A—H4A	119.5	C56B—C4B—H4BA	119.8
C4A—C5A—C6A	121.6 (4)	C1B—C6B—C56B	116.5 (4)
C4A—C5A—H5A	119.2	C1B—C6B—C7B	122.0 (4)
C6A—C5A—H5A	119.2	C56B—C6B—C7B	121.5 (4)
C1A—C6A—C5A	117.5 (4)	C8B—C7B—C12B	118.0 (4)
C1A—C6A—C7A	121.8 (4)	C8B—C7B—C6B	121.3 (4)
C5A—C6A—C7A	120.7 (4)	C12B—C7B—C6B	120.6 (4)
C8A—C7A—C12A	116.6 (4)	C7B—C8B—C9B	120.9 (5)
C8A—C7A—C6A	121.9 (4)	C7B—C8B—H8BA	119.6
C12A—C7A—C6A	121.4 (4)	C9B—C8B—H8BA	119.6
C9A—C8A—C7A	122.0 (4)	C10B—C9B—C8B	120.2 (6)
C9A—C8A—H8A	119.0	C10B—C9B—H9BA	119.9
C7A—C8A—H8A	119.0	C8B—C9B—H9BA	119.9
C8A—C9A—C10A	120.0 (4)	C9B—C10B—C11B	119.8 (5)
C8A—C9A—H9A	120.0	C9B—C10B—H10B	120.1
C10A—C9A—H9A	120.0	C11B—C10B—H10B	120.1
C11A—C10A—C9A	118.8 (5)	C10B—C11B—C12B	120.0 (5)
C11A—C10A—H10A	120.6	C10B—C11B—H11B	120.0
C9A—C10A—H10A	120.6	C12B—C11B—H11B	120.0
C12A—C11A—C10A	121.5 (5)	C11B—C12B—C7B	121.1 (5)
C12A—C11A—H11A	119.3	C11B—C12B—H12B	119.5
C10A—C11A—H11A	119.3	C7B—C12B—H12B	119.5
C11A—C12A—C7A	121.1 (5)	C14B—C13B—C18B	121.8 (4)
C11A—C12A—H12A	119.4	C14B—C13B—H13B	119.1
C7A—C12A—H12A	119.4	C18B—C13B—H13B	119.1
C14A—C13A—C18A	120.7 (4)	C15B—C14B—C13B	120.4 (4)
C14A—C13A—H13A	119.6	C15B—C14B—H14B	119.8
C18A—C13A—H13A	119.6	C13B—C14B—H14B	119.8
C13A—C14A—C15A	122.1 (4)	C14B—C15B—C16B	118.5 (4)
C13A—C14A—H14A	119.0	C14B—C15B—As1B	122.3 (4)
C15A—C14A—H14A	119.0	C16B—C15B—As1B	118.5 (3)
C14A—C15A—C16A	117.0 (4)	C17B—C16B—C15B	121.2 (4)
C14A—C15A—As1A	118.3 (3)	C17B—C16B—H16B	119.4
C16A—C15A—As1A	124.6 (4)	C15B—C16B—H16B	119.4
C17A—C16A—C15A	121.0 (4)	C16B—C17B—C18B	120.8 (4)
C17A—C16A—H16A	119.5	C16B—C17B—H17B	119.6
C15A—C16A—H16A	119.5	C18B—C17B—H17B	119.6
C16A—C17A—C18A	121.7 (4)	C13B—C18B—C17B	117.2 (4)
C16A—C17A—H17A	119.2	C13B—C18B—C19B	120.1 (4)
C18A—C17A—H17A	119.2	C17B—C18B—C19B	122.7 (4)
C17A—C18A—C13A	117.3 (4)	C24B—C19B—C20B	117.4 (4)
C17A—C18A—C19A	122.4 (4)	C24B—C19B—C18B	120.9 (4)
C13A—C18A—C19A	120.3 (4)	C20B—C19B—C18B	121.7 (4)
C20A—C19A—C24A	117.7 (4)	C21B—C20B—C19B	121.0 (4)
C20A—C19A—C18A	120.7 (4)	C21B—C20B—H20B	119.5
C24A—C19A—C18A	121.6 (4)	C19B—C20B—H20B	119.5
C19A—C20A—C21A	120.4 (5)	C20B—C21B—C22B	120.0 (5)
C19A—C20A—H20A	119.8	C20B—C21B—H21B	120.0
C21A—C20A—H20A	119.8	C22B—C21B—H21B	120.0
C22A—C21A—C20A	120.4 (5)	C23B—C22B—C21B	120.2 (5)
C22A—C21A—H21A	119.8	C23B—C22B—H22B	119.9
C20A—C21A—H21A	119.8	C21B—C22B—H22B	119.9
C23A—C22A—C21A	119.4 (5)	C22B—C23B—C24B	119.5 (4)
C23A—C22A—H22A	120.3	C22B—C23B—H23B	120.2
C21A—C22A—H22A	120.3	C24B—C23B—H23B	120.2
C22A—C23A—C24A	120.5 (5)	C19B—C24B—C23B	121.8 (4)
C22A—C23A—H23A	119.8	C19B—C24B—H24B	119.1
C24A—C23A—H23A	119.8	C23B—C24B—H24B	119.1
C23A—C24A—C19A	121.6 (4)	C26B—C25B—C30B	121.4 (4)
C23A—C24A—H24A	119.2	C26B—C25B—H25B	119.3
C19A—C24A—H24A	119.2	C30B—C25B—H25B	119.3
C26A—C25A—C30A	121.4 (4)	C25B—C26B—C27B	119.7 (4)
C26A—C25A—H25A	119.3	C25B—C26B—H26B	120.1
C30A—C25A—H25A	119.3	C27B—C26B—H26B	120.1
C25A—C26A—C27A	122.1 (4)	C28B—C27B—C26B	118.9 (4)
C25A—C26A—H26A	118.9	C28B—C27B—As1B	116.9 (3)
C27A—C26A—H26A	118.9	C26B—C27B—As1B	123.7 (3)
C26A—C27A—C28A	116.2 (4)	C29B—C28B—C27B	120.7 (4)
C26A—C27A—As1A	115.7 (4)	C29B—C28B—H28B	119.6
C28A—C27A—As1A	127.9 (3)	C27B—C28B—H28B	119.6
C29A—C28A—C27A	122.1 (4)	C28B—C29B—C30B	120.9 (4)
C29A—C28A—H28A	118.9	C28B—C29B—H29B	119.5
C27A—C28A—H28A	118.9	C30B—C29B—H29B	119.5
C28A—C29A—C30A	121.3 (4)	C29B—C30B—C25B	118.3 (4)
C28A—C29A—H29A	119.3	C29B—C30B—C31B	120.0 (4)
C30A—C29A—H29A	119.3	C25B—C30B—C31B	121.7 (4)
C25A—C30A—C29A	116.6 (4)	C32B—C31B—C36B	117.1 (4)
C25A—C30A—C31A	121.5 (4)	C32B—C31B—C30B	121.7 (4)
C29A—C30A—C31A	121.8 (4)	C36B—C31B—C30B	121.1 (4)
C32A—C31A—C36A	116.5 (4)	C31B—C32B—C33B	121.0 (4)
C32A—C31A—C30A	121.7 (4)	C31B—C32B—H32B	119.5
C36A—C31A—C30A	121.8 (4)	C33B—C32B—H32B	119.5
C31A—C32A—C33A	122.2 (4)	C34B—C33B—C32B	120.2 (5)
C31A—C32A—H32A	118.9	C34B—C33B—H33B	119.9
C33A—C32A—H32A	118.9	C32B—C33B—H33B	119.9
C34A—C33A—C32A	119.3 (5)	C33B—C34B—C35B	119.6 (5)
C34A—C33A—H33A	120.3	C33B—C34B—H34B	120.2
C32A—C33A—H33A	120.3	C35B—C34B—H34B	120.2
C35A—C34A—C33A	119.4 (4)	C36B—C35B—C34B	119.9 (5)
C35A—C34A—H34A	120.3	C36B—C35B—H35B	120.0
C33A—C34A—H34A	120.3	C34B—C35B—H35B	120.0
C34A—C35A—C36A	121.1 (4)	C35B—C36B—C31B	122.1 (5)
C34A—C35A—H35A	119.5	C35B—C36B—H36B	118.9
C36A—C35A—H35A	119.5	C31B—C36B—H36B	118.9
C35A—C36A—C31A	121.5 (4)	C4B—C56B—C6B	122.0 (5)
C35A—C36A—H36A	119.2	C4B—C56B—H56A	119.0
C31A—C36A—H36A	119.2	C6B—C56B—H56A	119.0
			
C6A—C1A—C2A—C3A	0.7 (7)	C6B—C1B—C2B—C3B	0.9 (8)
C1A—C2A—C3A—C4A	−2.3 (7)	C1B—C2B—C3B—C4B	0.9 (8)
C1A—C2A—C3A—As1A	−176.1 (3)	C1B—C2B—C3B—As1B	−175.8 (4)
C27A—As1A—C3A—C2A	−151.9 (3)	C27B—As1B—C3B—C4B	−70.3 (5)
C15A—As1A—C3A—C2A	−48.9 (4)	C15B—As1B—C3B—C4B	31.9 (5)
C27A—As1A—C3A—C4A	34.7 (4)	C27B—As1B—C3B—C2B	106.1 (4)
C15A—As1A—C3A—C4A	137.8 (4)	C15B—As1B—C3B—C2B	−151.6 (4)
C2A—C3A—C4A—C5A	2.2 (6)	C2B—C3B—C4B—C56B	−1.8 (8)
As1A—C3A—C4A—C5A	175.6 (3)	As1B—C3B—C4B—C56B	174.6 (4)
C3A—C4A—C5A—C6A	−0.3 (6)	C2B—C1B—C6B—C56B	−1.7 (7)
C2A—C1A—C6A—C5A	1.2 (6)	C2B—C1B—C6B—C7B	177.7 (4)
C2A—C1A—C6A—C7A	−178.2 (4)	C1B—C6B—C7B—C8B	174.3 (5)
C4A—C5A—C6A—C1A	−1.4 (6)	C56B—C6B—C7B—C8B	−6.3 (7)
C4A—C5A—C6A—C7A	178.1 (4)	C1B—C6B—C7B—C12B	−5.1 (7)
C1A—C6A—C7A—C8A	−148.3 (4)	C56B—C6B—C7B—C12B	174.3 (5)
C5A—C6A—C7A—C8A	32.2 (6)	C12B—C7B—C8B—C9B	−1.2 (8)
C1A—C6A—C7A—C12A	34.2 (7)	C6B—C7B—C8B—C9B	179.3 (5)
C5A—C6A—C7A—C12A	−145.3 (5)	C7B—C8B—C9B—C10B	0.7 (9)
C12A—C7A—C8A—C9A	−1.4 (7)	C8B—C9B—C10B—C11B	−0.1 (8)
C6A—C7A—C8A—C9A	−179.0 (4)	C9B—C10B—C11B—C12B	−0.1 (8)
C7A—C8A—C9A—C10A	0.0 (7)	C10B—C11B—C12B—C7B	−0.4 (8)
C8A—C9A—C10A—C11A	1.6 (8)	C8B—C7B—C12B—C11B	1.1 (7)
C9A—C10A—C11A—C12A	−1.8 (8)	C6B—C7B—C12B—C11B	−179.5 (4)
C10A—C11A—C12A—C7A	0.4 (9)	C18B—C13B—C14B—C15B	0.9 (7)
C8A—C7A—C12A—C11A	1.2 (8)	C13B—C14B—C15B—C16B	1.9 (6)
C6A—C7A—C12A—C11A	178.8 (5)	C13B—C14B—C15B—As1B	−168.8 (3)
C18A—C13A—C14A—C15A	0.8 (7)	C3B—As1B—C15B—C14B	−124.3 (4)
C13A—C14A—C15A—C16A	−3.7 (6)	C27B—As1B—C15B—C14B	−22.4 (4)
C13A—C14A—C15A—As1A	173.2 (3)	C3B—As1B—C15B—C16B	64.9 (4)
C3A—As1A—C15A—C14A	107.7 (4)	C27B—As1B—C15B—C16B	166.8 (4)
C27A—As1A—C15A—C14A	−147.1 (4)	C14B—C15B—C16B—C17B	−2.7 (7)
C3A—As1A—C15A—C16A	−75.6 (4)	As1B—C15B—C16B—C17B	168.4 (3)
C27A—As1A—C15A—C16A	29.6 (4)	C15B—C16B—C17B—C18B	0.6 (7)
C14A—C15A—C16A—C17A	2.3 (6)	C14B—C13B—C18B—C17B	−2.9 (6)
As1A—C15A—C16A—C17A	−174.4 (3)	C14B—C13B—C18B—C19B	175.7 (4)
C15A—C16A—C17A—C18A	2.0 (6)	C16B—C17B—C18B—C13B	2.1 (6)
C16A—C17A—C18A—C13A	−4.9 (6)	C16B—C17B—C18B—C19B	−176.4 (4)
C16A—C17A—C18A—C19A	173.6 (4)	C13B—C18B—C19B—C24B	−27.3 (6)
C14A—C13A—C18A—C17A	3.5 (6)	C17B—C18B—C19B—C24B	151.1 (4)
C14A—C13A—C18A—C19A	−175.1 (4)	C13B—C18B—C19B—C20B	153.7 (4)
C17A—C18A—C19A—C20A	−27.3 (7)	C17B—C18B—C19B—C20B	−27.8 (6)
C13A—C18A—C19A—C20A	151.2 (5)	C24B—C19B—C20B—C21B	−0.4 (6)
C17A—C18A—C19A—C24A	154.8 (4)	C18B—C19B—C20B—C21B	178.6 (4)
C13A—C18A—C19A—C24A	−26.8 (6)	C19B—C20B—C21B—C22B	0.2 (7)
C24A—C19A—C20A—C21A	−0.2 (8)	C20B—C21B—C22B—C23B	0.6 (7)
C18A—C19A—C20A—C21A	−178.3 (5)	C21B—C22B—C23B—C24B	−1.1 (7)
C19A—C20A—C21A—C22A	−0.8 (10)	C20B—C19B—C24B—C23B	−0.2 (6)
C20A—C21A—C22A—C23A	0.5 (10)	C18B—C19B—C24B—C23B	−179.1 (4)
C21A—C22A—C23A—C24A	0.7 (9)	C22B—C23B—C24B—C19B	0.9 (7)
C22A—C23A—C24A—C19A	−1.7 (8)	C30B—C25B—C26B—C27B	0.1 (6)
C20A—C19A—C24A—C23A	1.4 (7)	C25B—C26B—C27B—C28B	−1.3 (6)
C18A—C19A—C24A—C23A	179.5 (4)	C25B—C26B—C27B—As1B	−173.0 (3)
C30A—C25A—C26A—C27A	0.0 (6)	C3B—As1B—C27B—C28B	−178.3 (4)
C25A—C26A—C27A—C28A	2.2 (6)	C15B—As1B—C27B—C28B	77.6 (4)
C25A—C26A—C27A—As1A	−173.5 (3)	C3B—As1B—C27B—C26B	−6.4 (4)
C3A—As1A—C27A—C26A	−149.4 (3)	C15B—As1B—C27B—C26B	−110.5 (4)
C15A—As1A—C27A—C26A	108.8 (3)	C26B—C27B—C28B—C29B	2.5 (6)
C3A—As1A—C27A—C28A	35.5 (4)	As1B—C27B—C28B—C29B	174.8 (3)
C15A—As1A—C27A—C28A	−66.3 (4)	C27B—C28B—C29B—C30B	−2.6 (7)
C26A—C27A—C28A—C29A	−1.8 (6)	C28B—C29B—C30B—C25B	1.3 (7)
As1A—C27A—C28A—C29A	173.2 (3)	C28B—C29B—C30B—C31B	−178.2 (4)
C27A—C28A—C29A—C30A	−0.8 (6)	C26B—C25B—C30B—C29B	−0.1 (7)
C26A—C25A—C30A—C29A	−2.5 (6)	C26B—C25B—C30B—C31B	179.5 (4)
C26A—C25A—C30A—C31A	180.0 (4)	C29B—C30B—C31B—C32B	31.7 (6)
C28A—C29A—C30A—C25A	2.9 (6)	C25B—C30B—C31B—C32B	−147.8 (4)
C28A—C29A—C30A—C31A	−179.6 (4)	C29B—C30B—C31B—C36B	−145.6 (4)
C25A—C30A—C31A—C32A	−174.5 (4)	C25B—C30B—C31B—C36B	34.9 (6)
C29A—C30A—C31A—C32A	8.1 (6)	C36B—C31B—C32B—C33B	0.8 (6)
C25A—C30A—C31A—C36A	4.9 (6)	C30B—C31B—C32B—C33B	−176.6 (4)
C29A—C30A—C31A—C36A	−172.5 (4)	C31B—C32B—C33B—C34B	−0.3 (6)
C36A—C31A—C32A—C33A	1.8 (6)	C32B—C33B—C34B—C35B	−0.5 (6)
C30A—C31A—C32A—C33A	−178.8 (4)	C33B—C34B—C35B—C36B	0.8 (7)
C31A—C32A—C33A—C34A	−2.1 (7)	C34B—C35B—C36B—C31B	−0.3 (7)
C32A—C33A—C34A—C35A	0.9 (7)	C32B—C31B—C36B—C35B	−0.5 (6)
C33A—C34A—C35A—C36A	0.6 (6)	C30B—C31B—C36B—C35B	176.9 (4)
C34A—C35A—C36A—C31A	−0.9 (7)	C3B—C4B—C56B—C6B	1.0 (8)
C32A—C31A—C36A—C35A	−0.3 (6)	C1B—C6B—C56B—C4B	0.7 (8)
C30A—C31A—C36A—C35A	−179.6 (4)	C7B—C6B—C56B—C4B	−178.7 (5)

### Hydrogen-bond geometry (Å, °)

**Table:**

Cg1, Cg2, Cg3, Cg4, Cg5, Cg6 and Cg7 are the centroids of the C25B–C30B, C19B–C24B, C7B–C12B, C31A–C36A, C1A–C6A, C31B–C36B and C13B–C18B benzene rings, respectively.

**Table:**

*D*—H···*A*	*D*—H	H···*A*	*D*···*A*	*D*—H···*A*
C4B—H4BA···Cg1^i^	0.93	2.77	3.583 (6)	147
C9A—H9A···Cg2^ii^	0.93	2.99	3.872 (6)	160
C11A—H11A···Cg3^iii^	0.93	2.88	3.723 (7)	153
C13B—H13B···Cg4	0.93	2.79	3.596 (5)	147
C14A—H14A···Cg5^i^	0.93	2.93	3.834 (5)	172
C24A—H24A···Cg6^iv^	0.93	2.70	3.582 (6)	160
C28B—H28B···Cg7^v^	0.93	2.56	3.313 (6)	139
C36A—H36A···Cg3^vi^	0.93	2.96	3.651 (6)	133

Symmetry codes: (i) *x*, *y*+1, *z*; (ii) *x*, −*y*, *z*+1/2; (iii) *x*−1, −*y*, *z*+1/2;  (iv) *x*−1, *y*+2, *z*; (v) *x*, *y*−1, *z*; (vi) *x*, −*y*+1, *z*+1/2.
